# Arbuscular mycorrhizal diversity increases across a plant productivity gradient driven by soil nitrogen availability

**DOI:** 10.1002/pei3.70002

**Published:** 2024-08-10

**Authors:** Morgan R. McPherson, Donald R. Zak, Inés Ibáñez, Rima A. Upchurch, William A. Argiroff

**Affiliations:** ^1^ School for Environment and Sustainability University of Michigan Ann Arbor Michigan USA; ^2^ Department of Ecology and Evolutionary Biology University of Michigan Ann Arbor Michigan USA

**Keywords:** *Acer rubrum*, *Acer saccharum*, arbuscular mycorrhizal fungi (AMF), basal area increment (BAI), *Glomus*, net N mineralization, temperate forest ecosystem

## Abstract

Arbuscular mycorrhizal fungi (AMF) are widespread obligate symbionts of plants. This dynamic symbiosis plays a large role in successful plant performance, given that AMF help to ameliorate plant responses to abiotic and biotic stressors. Although the importance of this symbiosis is clear, less is known about what may be driving this symbiosis, the plant's need for nutrients or the excess of plant photosynthate being transferred to the AMF, information critical to assess the functionality of this relationship. Characterizing the AMF community along a natural plant productivity gradient is a first step in understanding how this symbiosis may vary across the landscape. We surveyed the AMF community diversity at 12 sites along a plant productivity gradient driven by soil nitrogen availability. We found that AMF diversity in soil environmental DNA significantly increased along with the growth of the host plants *Acer* *rubrum* and *A. saccharum*., a widespread tree genus. These increases also coincided with a natural soil inorganic N availability gradient. We hypothesize photosynthate from the increased tree growth is being allocated to the belowground AMF community, leading to an increase in diversity. These findings contribute to understanding this complex symbiosis through the lens of AMF turnover and suggest that a more diverse AMF community is associated with increased host–plant performance.

## 1 INTRODUCTION

**FIGURE 1:**
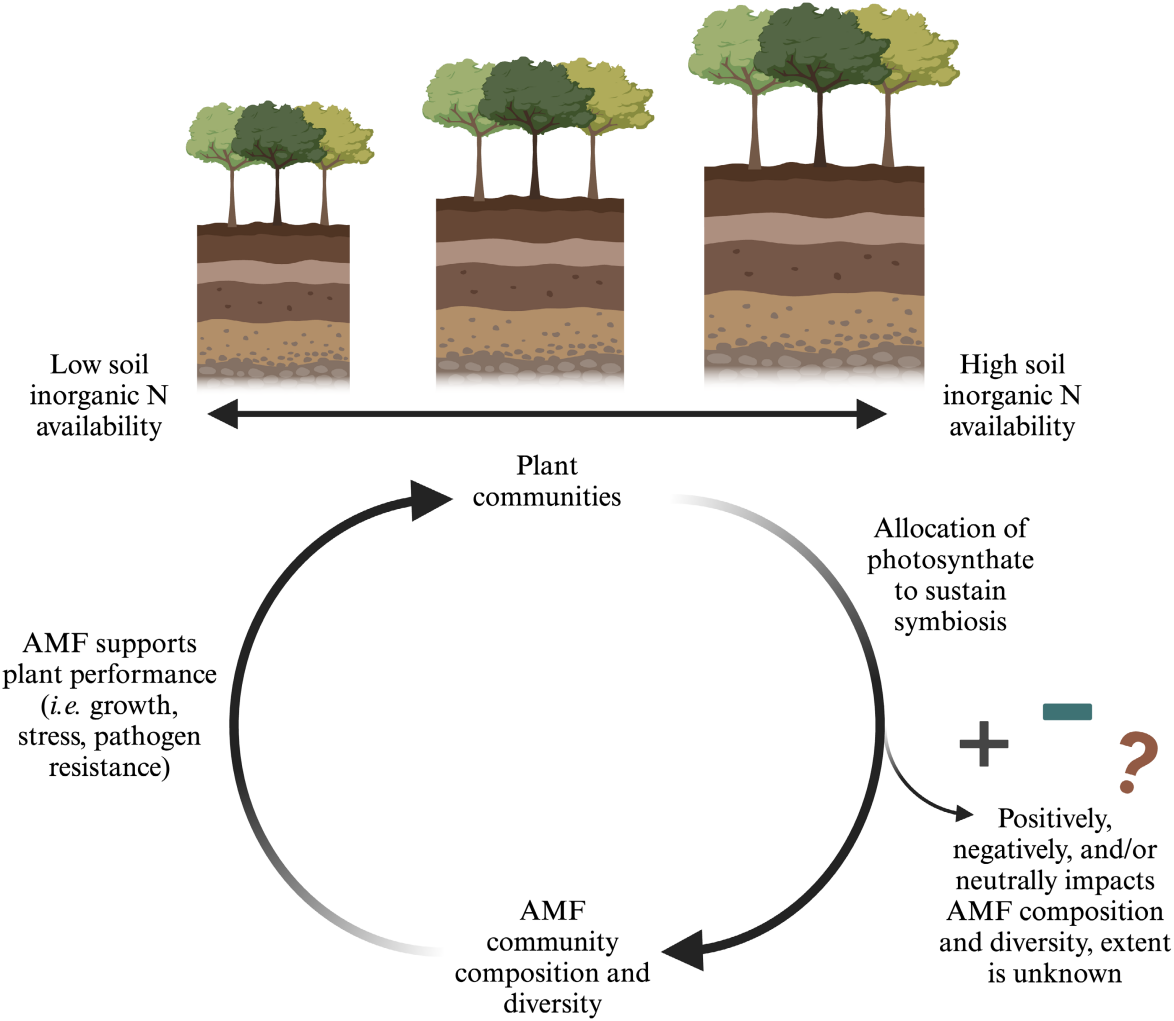
Conceptual diagram displaying the interplay between the natural resource availability gradient and increasing plant growth over the gradient, plant communities, AMF community composition and diversity, and plant performance. Image created with BioRender.com.

Arbuscular mycorrhizal fungi (AMF) are obligate symbionts that associate with the roots of many terrestrial plants (Smith & Read, 2008; Van Der Heijden et al., 2015). The AMF symbiosis is ancient and provides biotic and abiotic advantages to terrestrial plants, such as increased access to water and nutrients as well as protection against pathogens (Lanfranco et al., 2016). Through this symbiosis, the plant host provides up to 20% of the photosynthetically fixed carbon to the symbiont, which sustains their ability to benefit host plant performance (Brundrett & Tedersoo, 2018; Roth & Paszkowski, 2017; Smith et al., 2011; Smith & Read, 2008). As a result, differences in plant productivity could shape the composition and diversity of AMF communities (Figure 1) by influencing the amount of photosynthate allocated belowground. Although the nature of this reciprocal relationship is clear, we understand little regarding changes in AMF community composition along gradients of plant productivity. Do shifts in AMF composition and diversity result from plant nutrient demand, greater amounts of photosynthate allocated belowground to AMF symbionts, or some combination of both? Furthermore, gaining insight into how AMF community composition and diversity changes along plant productivity gradients may reveal concomitant changes in AMF community function. To address this knowledge gap, we examined the diversity of AMF communities associated with individual trees along a naturally occurring gradient of plant productivity. As Earth System Models (ESM) aim to incorporate the role of the mycorrhizal communities on plant productivity, understanding this knowledge gap becomes critical for ensuring accurate predictions of terrestrial productivity and carbon storage (Reich et al., 2006; Terrer et al., 2019; Wieder et al., 2015).

Arbuscular mycorrhizal fungi community composition and diversity have variable impacts on host plant performance. For example, the interactions between AMF and the host plant can range from mutualism to antagonism, depending on the specific environmental conditions (Ibáñez & McCarthy‐Neumann, 2016; Wang et al., 2023); however, AMF are mostly known for their role in facilitating nutrient uptake, specifically nitrogen (N) and phosphorus (P) (Wang et al., 2023; Xie et al., 2022). Nitrogen is acquired by plants through the mycorrhizal interface, wherein AMF transfer inorganic N to the host using high affinity nutrient transporters in the extraradical mycelium (ERM) (Bücking & Kafle, 2015; Hodge & Fitter, 2010). Increased plant performance resulting AMF symbioses has been documented in a wide range of environments as well as among plant taxa (Diagne et al., 2020; Nakmee et al., 2016). Despite these observations, positive, negative, and neutral responses of plant performance to AMF have occurred, suggesting that the benefit or cost of AMF can be dependent on multiple environmental factors (Egerton‐Warburton et al., 2007). Presently, there is ambiguity in how these symbioses function, i.e., are they driven by plant nutrient demand, by the belowground allocation of photosynthate to AMF, or both?

Under similar environmental conditions, such as climate and soil type, plant productivity is usually driven by soil resource availability (i.e., soil N availability; Ordoñez et al., 2009; Rennenberg et al., 2009). Despite greater amounts of photosynthate available for belowground allocation when soil resources are abundant, there is likely a decreasing benefit of the AMF symbiosis to the host plant (Ma et al., 2021); the greatest benefit to plant performance appears to occur in nutrient‐poor soil (Bertolazi et al., 2018). As a result, AMF community composition, diversity, and function could change as plant productivity increases across a soil nutrient gradient. In a natural grassland experiment, AMF species richness was positively correlated with plant belowground richness and plant community biomass (Hiiesalu et al., 2014), suggesting that plant excess photosynthate, and not plant nutrient demand, shape AMF richness.

Soil pH and moisture are two of the main environmental drivers of AMF community composition and diversity (Dumbrell et al., 2010). The range of soil pH limits mycorrhizal colonization and subsequent growth, which in turn impacts the overall community composition (Chen et al., 2022). AMF communities also are sensitive to soil moisture, because AMF spores in the soil require moisture to germinate and colonize the plant roots, as well as support extraradical mycelial growth (Wang et al., 2021). Finally, soil texture impacts AMF community diversity as AMF function can shift with varying textures (Lekberg et al., 2007). Therefore, disentangling how AMF community composition and diversity vary along gradients of plant productivity needs to account for potentially covarying edaphic conditions.

Insights into this interplay may be gained by studying the composition and diversity of AMF communities of a single host plant that occur across a gradient of plant productivity under similar climatic and soil conditions. Using a naturally occurring gradient of soil N availability along which plant growth increases, we quantified the composition, community turnover, and diversity of AMF on a single host tree genus (*Acer rubrum* and *A. saccharum*). We sampled, processed, and sequenced soil samples directly from individual trees across a net N mineralization gradient. This gradient has remained stable over time (Argiroff et al., 2022; Pellitier, Ibáñez et al., 2021; Pellitier, Zak et al., 2021; Zak et al., 1989; Zak & Pregitzer, 1990) and is well‐suited to provide insight into plant–mycorrhizal relationships, especially given that differences in climate, soil texture, and other physical properties are minimal across sites. Using this approach, we cannot discern causation in the bi‐directional interaction between a host plant and its AMF community, but as a first step, we can reveal patterns in AMF diversity along a natural soil resource availability gradient that is linked to plant performance. Specifically, our objective was to evaluate the diversity of AMF communities of individual trees along a naturally occurring gradient of soil inorganic N availability in northern broadleaf temperate forests. Understanding the diversity of AMF communities along naturally occurring plant productivity gradient could shed light on predicting potential function of AMF in fostering plant growth, which has now been incorporated into global biogeochemical models (Terrer et al., 2018) predicting terrestrial productivity and carbon storage.

## 2 MATERIALS AND METHODS

### 2.1 Site descriptions and study design

We sampled the soil around 42 *Acer rubrum* (red maple) and 30 *A. saccharum* (sugar maple) trees across a natural soil inorganic N availability gradient in the Manistee National Forest in northern Lower Michigan, USA (Figure S1; gradient spans ~76 km). Trees were located in 12 even‐aged second growth stands (sites) that have regenerated following clear‐cutting in the early 20th century and have been previously described (Argiroff et al., 2022; Pellitier, Zak et al., 2021; Zak et al., 1986). Both maple species are common and widely distributed across eastern North America, and broadly occur within these northern hardwood forests. Dominant overstory individual within each site were selected for this study. *Acer rubrum* is known to thrive on diverse sites, from dry ridges and southwest slopes to peat bogs and swamps, and it is moderately shade tolerant (Burns & Honkala, 1990). *Acer saccharum* is not as widely distributed as *A. rubrum*, and grows on mesic and well‐drained soil; it is shade tolerant and slow growing (Burns & Honkala, 1990). Across our study sites, N availability ranges from ~80 to 120 kg N ha^−1^ year^−1^, which captures the full range of N availability in the upper Lake States region (Zak & Pregitzer, 1990). Variation in inorganic N availability that creates the natural gradient has arisen from the effects of physiography on microclimate and nutrient retention over the past 10,000 years, and this gradient has remained seasonally and inter‐annually stable (Pellitier, Ibáñez et al., 2021; Pellitier, Zak et al., 2021; Zak et al., 1989). Soil texture is uniform (~85% sand; Zak et al., 1989) and macroclimate (i.e., annual precipitation and temperature) does not differ due to close geographic proximity among stands (separated by <50 km).

### 2.2 Tree sampling

In June 2022, at each site, we identified *Acer* individuals larger than 10 cm diameter at breast height (1.3 m; DBH) within each forest stand. We extracted tree cores from five individuals at DBH using Haglöf 5.15 mm increment borers (Haglöf Inc.). The north and south side of each tree was cored to the pith, and cores were stored in paper straws until they could be dried. We dried core samples overnight in a 100°C oven and mounted them afterwards in cradles. Cores were then sanded with progressively finer sandpapers starting at 100 grit and ending at 1600 grit. We digitized the prepared samples using a flatbed scanner at a resolution of 1200 dpi.

We measured annual ring width (growth) of digitized scans at a precision of 0.001 mm using the Cybis CooRecorder program. We then used Cybis CDendro to crossdate samples and assemble different chronologies by site and species, estimating pith using the geometric method if not present on the sample (Duncan, 1989). Correlations among chronologies (expressed population signal, EPS) vary between 0.57 and 0.79 for *A. rubrum* and 0.42 and 0.78 for *A. saccharum*. We estimated historical DBH for tree *i* in year *y* using ring width and the diameter of trees in 2022. To measure yearly growth for use in our analyses, we calculated Basal Area Increment (BAI) as:

(1) $$\mathrm{BAI}i,y=\pi ∙{\frac{{\mathrm{DBH}}_{i,y}}{4}}^{2}-{\frac{{\mathrm{DBH}}_{i,y-1}}{4}}^{2}$$

### 2.3 Tree core analysis

Tree basal area increment (BAI) estimates of each tree from 1981 to 2021 were analyzed as a function of N mineralization (*N*min) in the soil collected around each tree, and as a function of other variables known to be associated with aboveground tree growth in these species, size (lnDBH) (Bigelow et al., 2021; Cook, 1990; Duncan, 1989), growth the previous year (BAI_*i,t‐1*_; a lag effect; Wang & Ibáñez, 2022), minimum temperature in May (*minMay*; a proxy for beginning, and length, of the growing season; Ibáñez et al., 2018; Pellitier, Ibáñez et al., 2021; Pellitier, Zak et al., 2021). We also included spatially explicit random effects (*SERE*) to account for any spatial dependencies within each site not accounted for by the covariates. We tried several combinations of covariates and functions and described below the model with the best fit based on deviance information criterion (DIC; Spiegelhalter et al., 2002). For each species independently, we modeled BAI for tree *i* in year *y* (bai_*i,y*_) with a log‐normal likelihood:

(2) $$\mathrm{BAI}i,y\sim L\text{normal}D_{i,y},{\sigma^{2}}_{i,y}$$

And process model:

(3) $$\begin{array}{c} D_{i,y}=\alpha +\beta_{1}∙N\min_{i}+\beta_{2}∙{\mathrm{BAI}}_{i,y-1}+\beta_{3}\ln\left({\mathrm{DBH}}_{i}\right)\\ \;+\beta_{4}∙{\text{MayminTemp}}_{y}+{\text{SERE}}_{i} \end{array}$$

To account for an increase in variance as growth increases with a greater DBH, we estimated the variance (${\sigma^{2}}_{i,t}$) as a function of DBH (Lines et al., 2012): ${\sigma^{2}}_{i,t}=a+b∙\ln\left({\mathrm{DBH}}_{i,t}\right).$ Spatially explicit random effects for each tree *i* were estimated as a function of distance to neighbor *j* (Distance_*i,j*_) using a decay function:

(4) $$\begin{array}{c} {\text{SERE}}_{i}\sim \text{Exponential}\left(\sum_{j=1}^{\mathrm{No}.{\text{Neigh}}_{i}}e^{-\varphi \cdot {\text{Distance}}_{i,j}},\sigma_{\text{SERE}}^{2}\right) \end{array}$$

All model parameters were estimated within a Bayesian framework from non‐informative prior distributions, $\alpha \sim \text{Uniform}\left(0,5\right),$ $\beta_{*}\sim \text{Normal}\left(0,1\right),a\sim \text{logNormal}\left(1,0.001\right),$ $b\sim \text{Normal}\left(0,0.001\right),$ $\varphi =\exp\left(\theta \right)$, $\theta \sim \text{Normal}\left(0,1\right),$ and $\sigma_{\text{SERE}}^{2}\sim \text{Uniform}\left(0,10\right).$

Analyses were conducted using JAGS (Plummer, 2021) and the rjags package in R (R Core Team, 2023). We ran three MCMC chains for 10,000 iterations until convergence was reached. The posterior parameter means, standard deviations, and 95% credible intervals were then estimated across 50,000 iterations.

### 2.4 Soil sampling

At the time of tree core extraction, we collected soil cores in a 2‐m radius around each individual tree. After removing intact and partially decayed litter (Oi and Oe horizons), eight soil cores (5‐cm diameter) were collected at evenly spaced intervals to a depth of 5 cm around each individual tree. The cores were composited by individual tree and transported on ice to the laboratory. We passed the soils through a 2‐mm sieve and immediately stored a subsample at −80°C for characterization of AMF communities. We used two additional fresh subsamples for the determination of inorganic N availability (described below) and air‐dried the remainder.

### 2.5 Characterization of soil properties

We used 35‐day laboratory net N mineralization assays to re‐confirm soil inorganic N availability among our study sites (Vitousek et al., 1982; Zak et al., 1989). Specifically, we extracted inorganic N (NO_3_^−^ and NH_4_^+^) with 2 M KCl, then measured the initial and post‐incubation extracts using an AQ2 Discrete Analyzer (SEAL Analytical). Laboratory net N mineralization measurements are a robust representation of inorganic N availability, because they are strongly correlated with *in situ* net N mineralization rates across these forest ecosystems (Zak et al., 1989; Zak & Pregitzer, 1990). Soil pH was measured using a 1:1 ratio of air dried soil and deionized water, and C and N were determined using a CN analyzer (LECO) as previously described by (Argiroff et al., 2022).

### 2.6 Microbial community characterization

We extracted genomic DNA from four 0.25 g subsamples of soil from around each tree (*n* = 72) using the PowerLyzer PowerSoil DNA Isolation Kit (Qiagen) with bead beading at 3000 rpm for 30 s and following the manufacturer's protocol. All extracted DNA quality and quantity was checked using gel electrophoresis and the Quant‐iT PicoGreen kit method (Thermo Fisher Scientific). The 18S region was amplified using modified NS31 and AML2, well characterized primers for AMF, to contain barcodes and Illumina dual‐indexed primers (Table S1; Lee et al., 2008; Morgan & Egerton‐Warburton, 2017; Simon et al., 1992). All PCRs were performed in triplicate following a modified protocol using Phusion High Fidelity DNA Polymerase and master mix (New England BioLabs, Argiroff et al., 2022; Taylor et al., 2016). Each PCR contained 5 μL High Fidelity Phusion 5 × buffer, 0.7 μL each primer (10 μM initial concentration), 2 μL dNTPs (20 mmol^−1^ initial concentration of each dNTP), 2 μL of template DNA (DNA concentration ranged from 8.5 to 55 ng/μL) and 0.2 μL of Phusion High Fidelity DNA Polymerase (2000 U/mL) brought to a final volume of 25 μL with 14.4 μL molecular‐grade water. PCR conditions consisted of an initial denaturation step at 95°C for 5 min, followed by 30 cycles of the following: 30 s at 95°C, 60 s at 69°C and 45 s at 72°C followed by a final extension step of 72°C for 3 min. PCR libraries were sequenced with MiSeq 2 × 250 bp with v2 chemistry (Illumina) at the Advanced Genomics Core at the University of Michigan.

### 2.7 AMF community composition

We calculated amplicon sequence variants (ASVs; Callahan et al., 2017; Pauvert et al., 2019) using forward reads only as there was no overlap and previous studies have found that forward reads alone resolve AMF taxonomically (Davison et al., 2012; Morgan & Egerton‐Warburton, 2017). ASVs were created using the “DADA2” pipeline (Callahan et al., 2016; Rosen et al., 2012) with “cutadapt” (Martin, 2011) in R version 4.3.0 (R Core Team, 2023) and RStudio version 4.3.0 (Posit team, 2023). All reads were filtered and trimmed using the following parameters: manN = 0, truncLen = 240, maxEE = 1.75, trunQ = 2, minLen = 200. We then assigned taxonomy using a local blast environment with a modified MaarjAM database (Öpik et al., 2010). The modified MaarjAM (non‐type) database was edited to remove sequences with excessively short or long length and with any ambiguous bases. Taxonomic assignments were assigned and filtered using a bitscore of 300 or higher as small portions of the 18S region had a high percent identity but with only partial overlap, resulting in an incorrect assignment by percent ID or *e*‐value alone; this step also removed suspected non‐AMF reads. To assess AMF diversity across the N mineralization gradient, we used the shared ASV communities between both maple species (147 ASVs) and calculated the Chao1 richness index (Chao, 1984). The Chao1 index is an indicator of species richness in a sample and is widely used in microbial ecology for assessing community diversity (Hughes et al., 2001; Kim et al., 2017).

### 2.8 Phylogenetic analysis of microbial data

A multiple sequence alignment (MSA) was created using MAFFT with the L‐INS‐i method (Katoh, 2002). The MSA contained virtual taxa (VTs) assignments from representative Glomeromycota genera as well as our ASV reads. Next, we generated a maximum likelihood phylogenetic tree with 1000 bootstrap replicates. The phylogenetic trees were inferred using a GTR + G + I + Gamma4 model in MEGA11 (Tamura et al., 2021). The phylogenetic tree was visualized and annotated using iTOL (Figure S2; Letunic & Bork, 2021).

### 2.9 Statistical analyses of microbial data

The Chao1 diversity relationship to average BAI per tree over the past 41 years and net N mineralization (Figure 3) were assessed using linear regression. To assess the turnover of the dominant AMF community associated with our environmental gradients, we used “TITAN2” (Baker & King, 2010). ASVs and VTs with both purity and reliability ≥0.95 were considered significantly related to BAI or the N mineralization gradient (Baker & King, 2010). Altogether, the following packages were used, “tidyverse” package for data filtering and visualization (Wickham et al., 2019), “TITAN2” to relate ASV and VT abundances to continuously distributed variables (Baker & King, 2010), “decostand” from the “vegan” package for data transformation (Oksanen et al., 2022), and “phyloseq” and “Biostrings” (McMurdie & Holmes, 2013; Pagès et al., 2020) for amplicon sequencing data manipulation. All statistical analyses were performed in R.

## 3 RESULTS

### 3.1 Tree growth

**FIGURE 2:**
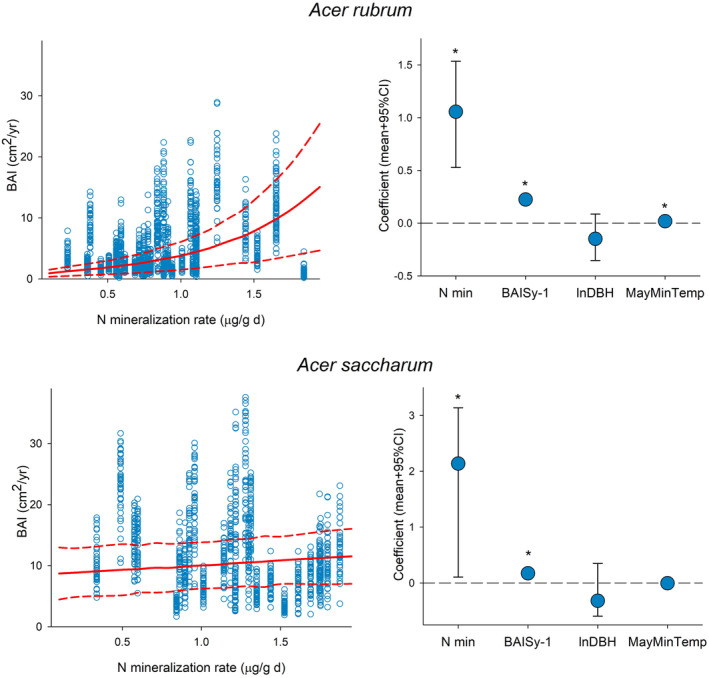
Growth data (dots) and predicted growth (lines) of *Acer rubrum* and A. *saccharum* increasing along the nitrogen mineralization gradient (left panels). Coefficients of the variables included in the analysis are net N mineralization rate in soil (Nmin), tree growth from the previous year (BAIS y‐1), tree size (lnDBH), and minimum temperature in May (MayMinTemp) (right panels). Asterisks indicate statistical significance; 95% credible intervals do not overlap with zero. Additional information is found in Table S3.

After discounting damaged tree cores we analyzed tree growth data for 38 *A. rubrum* individuals and 26 *A. saccharum* trees. Net N mineralization ranged from 0.22 to 1.84 μg g^−1^ day^−1^ for *A. rubrum* and from 0.34 to 1.87 μg g^−1^ day^−1^ for *A. saccharum* (Figure 2). As we expected, tree growth increased with increasing levels of N mineralization for the two species (Figure 2). The model goodness of fit (*R* ^2^) was 0.80 for *A. rubrum* and 0.76 for *A. saccharum*. Growth was also significantly associated with growth the previous year, and, in the case of *A. rubrum*, with higher May minimum temperatures (Figure 2). Parameter values of the analyses are found in the supplemental information (Table S3).

### 3.2 Characterization of AMF taxa and their variability across the N mineralization gradient

Our sequencing effort resulted in approximately 5 million reads. After quality control and removal of non‐AMF reads, we had 499 ASVs across all four orders within Glomeromycota. Taxonomic assignment to VTs with the MaarjAM database showed that 183 ASVs collapsed into 51 individual VTs within six genera (*Glomus, Paraglomus, Acaulospora, Diversispora*, *Claroideospora*, and *Archaeospora*). To verify taxonomic assignments, we constructed a phylogenetic tree that confirmed our sequences spanned all known orders of AMF (Figure S2). However, even with our sequencing coverage of Glomeromycota, the bulk of the sequences were assigned to the genus *Glomus* (Figure S2).

**FIGURE 3:**
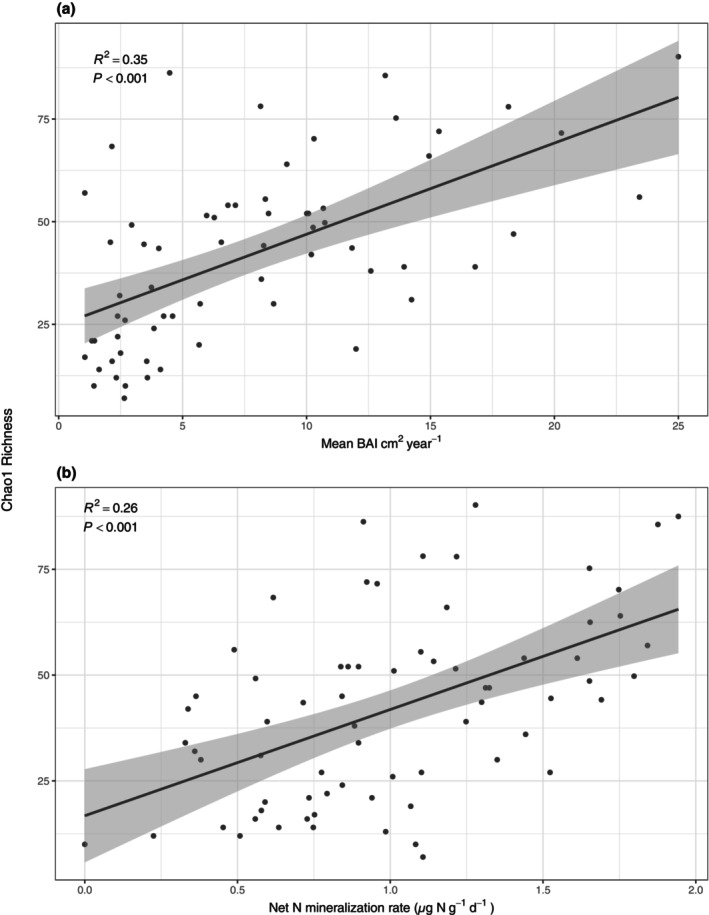
The relationship between Chao1 AMF species richness and the (a) mean BAI per tree over the last 41 years representative of *Acer rubrum* and *A. saccharum* growth and (b) the net N mineralization gradient. The plots represent significant regressions with Chao1 species richness as the dependent variable and (a) mean BAI and (b) N mineralization gradient as the independent variables, from which the adjusted R2 was calculated, the shaded area represents 95% confidence intervals.

To better understand the dominant members within the AMF community, we focused on the ASVs that occurred on multiple trees across sites and only those that appeared from both tree species which resulted in 171 ASVs total. The AMF species richness of this shared community significantly increased as tree growth, measured as average BAI per tree from the past 41 years, significantly increased (*F*_1,62_ = 34.2, *p* < 0.001, adj. *R* ^2^ = 0.35; Figure 3a). There was also a significant relationship to the net N mineralization rate (*F*_1,68_ = 25.3, *p* < 0.001, adj. *R* ^2^ = 0.26; Figure 3b).

**FIGURE 4:**
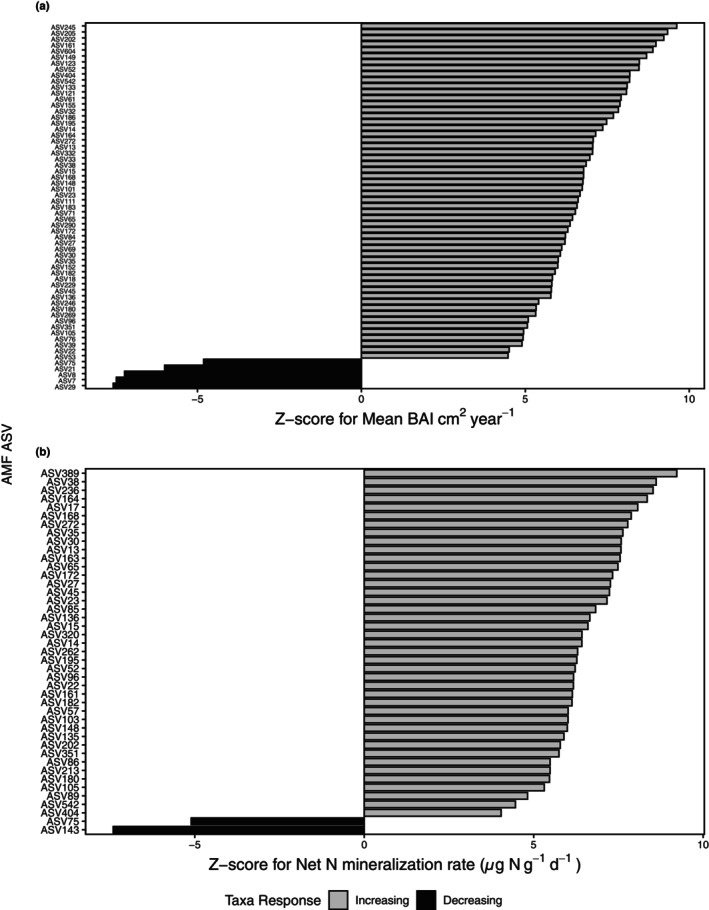
Responses to (a) tree growth measured as BAI and (b) inorganic N availability for individual AMF ASVs in soil determined by TITAN2 analysis. For BAI, 56 of our AMF ASVs increased, whereas only 5 decreased. For N mineralization, 41 ASVs increased, whereas 2 decreased. For both plots, bars display median Z‐scores (across 1000 bootstrap replicates), which represent the magnitude of the change in ASV relative abundance across BAI and the gradient of inorganic N availability, respectively. Positive Z‐scores indicate genera that increased with increasing BAI or inorganic N availability, whereas negative Z‐scores indicate genera that decreased in relative abundance with BAI or increasing inorganic N availability. We considered responses with both purity and reliability ≥0.95 as statistically significant. ASV abundances were Hellinger‐transformed prior to TITAN2 analysis and shared across both *Acer* spp.

**FIGURE 5:**
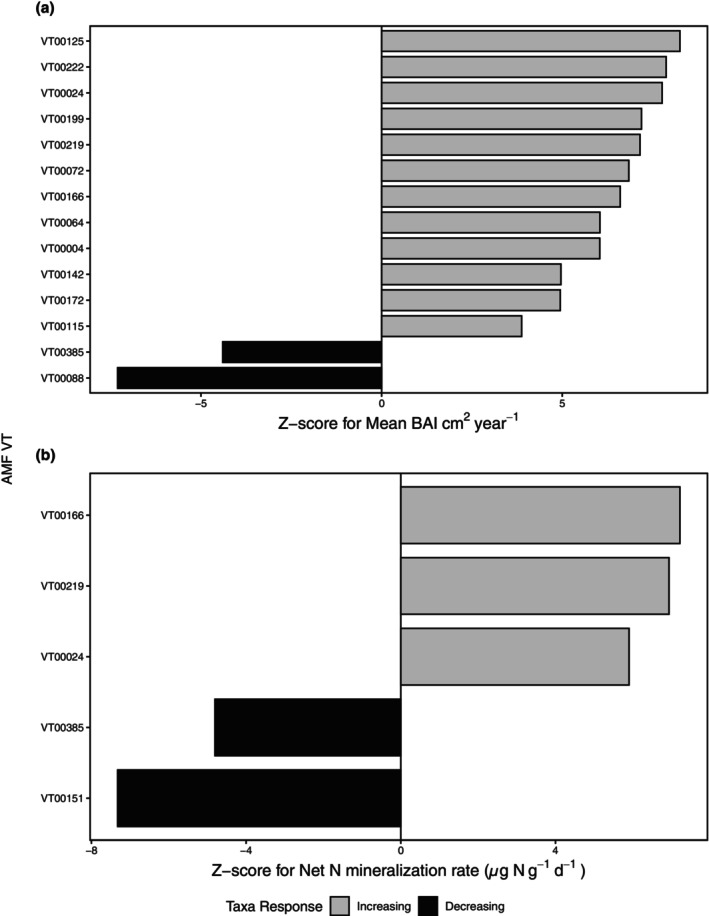
Responses to (a) tree growth measured as BAI and (b) inorganic N availability for individual virtual taxa (VTs) in soil determined by TITAN2 analysis. For BAI, 12 of our AMF VTs increased, whereas only 2 decreased. For N mineralization, 3 VTs increased, whereas 2 decreased. For both plots, bars display median Z‐scores (across 1000 bootstrap replicates), which represent the magnitude of the change in VT relative abundance across BAI and the gradient of inorganic N availability, respectively. Positive Z‐scores indicate VTs that increased with increasing BAI or inorganic N availability, whereas negative Z‐scores indicate VTs that decreased in relative abundance with BAI or increasing inorganic N availability. We considered responses with both purity and reliability ≥0.95 as statistically significant. VT abundances were Hellinger‐transformed prior to TITAN2 analysis and shared across both *Acer* spp.

Furthermore, to evaluate AMF community turnover along our environmental gradient, we used TITAN2 analysis. TITAN2 tested individual taxa, AMF ASVs and VTs, against the environmental gradients and two metrics, purity and reliability, were used to evaluate the strength of the relationship of that taxon to the gradients. We tested for AMF ASVs (Figure 4) and VTs (Figure 5) that significantly responded to BAI and the net N mineralization gradient, based on Hellinger‐transformed abundances (Legendre & Legendre, 2012). We found that 56 ASVs increased in relative abundance to plant growth measured as BAI, whereas only 5 decreased (Figure 4a). Similarly, 41 ASVs increased as N mineralization increased, whereas 2 ASVs decreased (Figure 4b). When all ASVs were collapsed into their respective VT assignments, we again examined these relationships between the relative abundance of the VTs compared with BAI and along the N mineralization gradient (Figure 5). We found that 12 VTs increased as BAI increased, whereas 2 decreased (Figure 5a), along with a similar relationship between N mineralization with 3 VTs that increased and 2 that decreased (Figure 5b). Overall, we see a significant increase in AMF ASVs, and corresponding taxonomic VTs, as plant growth (BAI) and inorganic N availability (N mineralization) significantly increase.

To highlight some of the prevalent VTs that appear in both increasing BAI and net N mineralization, VT00166 and VT00219 have previously been described as generalists and all significant VTs shown are prevalent at a global scale across most biomes (Davison et al., 2011; Djotan et al., 2023; Öpik et al., 2010; Větrovský et al., 2023). Both VT00166 and VT00219 have been previously observed on *Acer* spp. All VTs have previously been found in soil or on plant roots (Öpik et al., 2010) and VT00219 has been identified as an indicator species in higher nutrient treatments (Liu et al., 2021). VT00385, which significantly responded negatively to both BAI and inorganic N availability, is also found in multiple ecosystem types and was previously shown to decrease with higher ambient N (Van Diepen et al., 2011).

## 4 DISCUSSION

Soil resource availability (i.e., N) is a main driver of plant productivity, the accessibility to which is facilitated by the AMF symbiosis. In turn, plants contribute to the symbiosis with photosynthate. What is not clear is whether the composition and diversity of the AMF community, and thus its function, results from plant nutrient demand, by the supply of photosynthate belowground, or both (Figure 1). Although numerous studies have shown the impact of AMF on plant performance (Chitarra et al., 2016; Delavaux et al., 2017; Lanfranco et al., 2016; Smith & Read, 2008), we lack a clear understanding of how plant productivity may shape the composition, diversity, and function of AMF communities. By studying the AMF community along tree growth and a soil resource gradient, we sought to reveal the relationship between plant performance and AMF community composition and diversity. Although disentangling causative mechanisms in this relationship is difficult without experimentation, we observed a high level of species turnover and greater diversity of AMF communities that corresponded to increases in host plant productivity.

If plants at the high end of soil N availability gradients depend less on AMF to meet their nutrient demand, then it is possible that greater amounts of photosynthate are available for their AMF symbionts. More specifically, trees on the higher end of the nutrient gradient may produce and allocate more photosynthate belowground, leading to greater availability with less competition for these resources, and thus promotion of greater diversity within the AMF community. Overall, a more diverse AMF community appears to result from greater amounts of photosynthate, along with increased tree growth (Figures 2 and 3), being allocated to fine roots and potentially their AMF symbionts (Fellbaum et al., 2012, 2014). Whether these compositional shifts translate to functional change in the AMF community remains an open question.

Along our N mineralization gradient there were some shifts in soil pH as expected, and minimal changes in soil moisture, and texture (Table S2). Although soil pH varies across the gradient, values range from 3.6 to 5.4 among sites. Soil moisture varied from 6.7 to 18.6% across sites, and soil texture is consistent, ~90% sand, throughout all sites (Zak et al., 1989), eliminating it as a contributor to the increase in AMF diversity. Although these soil environmental factors play a part in shaping AMF community composition and diversity, our results suggest increased plant productivity and potentially more photosynthate, originating from increased soil N availability, are driving changes in AMF community diversity.

Overall, AMF composition and diversity within plant roots and the soil is highly variable and differs seasonally, annually, methodologically, by host plant, and by ecosystem type (Egerton‐Warburton et al., 2007; Helgason et al., 1998; Johnson et al., 2003; Treseder & Allen, 2002; Van Der Heijden et al., 1998). By controlling for many of these factors, we detected an increase in AMF richness along a soil N availability gradient, yet the main genus across all sites was *Glomus* (Figure S2). This increase in richness suggests an additive effect, wherein multiple AMF taxa, from the dominant *Glomus* along with other genera, increased together across the gradient to create an overall pattern of increased AMF diversity. We suspect this effect likely results from increases in plant productivity along the gradient, due to higher soil moisture and N availability, which would support the more diverse AMF community by allocating more photosynthate belowground. Previous research conducted in similar forests of this region found that AMF communities on *A. sacccharum* roots were also predominately colonized by *Glomus* and some *Acaulospora* (Van Diepen et al., 2011). In addition to this, other studies have reported a dominance of *Glomus* in the soil community. This may be due to the ability of this genus to survive and propagate more efficiently via pieces of mycelium or mycorrhizal root fragments (Daniell et al., 2001; Hart & Reader, 2002; Suzuki et al., 2020). Given the increase in AMF diversity as well as the increase in plant performance of *Acer* spp. across the gradient, we cannot discern whether this is being driven by either the plant or the AMF community; however, we can conclude that there is an additive effect occurring in terms of increasing AMF diversity.

It is important to acknowledge the challenges that arise from studying the interplay of this symbiosis (Figure 1). Specifically, drawing causation from the patterns that we have found proves especially difficult. Given these patterns of AMF diversity, which coincide with plant performance, we suggest that the next step is to examine the way the trees are contributing to the allocation or selection of the symbionts through the flux of photosynthate belowground. More specifically, which components, traits, or environmental factors of the plant community lead to a positive, negative, or neutral impact on the AMF community (Figure 1). Disentangling this portion (plant community influence of AMF community composition and diversity; Figure 1) of the bi‐directional symbiosis could lead to a more comprehensive understanding of the functionality of these interactions and its role in the carbon cycle.

In addition to this, there are ambiguities that come with translating AMF community composition and diversity to potential function, given current experimental tools and frameworks. Some of these ambiguities arise from the biology of AMF, more specifically that they are notoriously difficult to study as they are reliant on a plant host for photosynthate, whereas some host plants can survive with or without AMF symbionts. To note, few AMF species (i.e. *Rhizophagus* spp. and *Gigaspora* spp.) can be cultured in a lab outside of their natural habitat in root organ cultures, but these taxa are the exception (Kokkoris et al., 2024; Sugiura et al., 2020). It may also be possible that we are unable to fully infer function using classic taxonomic and phylogenetic approaches when studying AMF, as there may be details we are unable to capture within the intricate communication of this bi‐directional symbiosis. Regardless of these challenges, there are multiple experimental approaches that have been implemented to identify AMF and newer approaches to potentially infer function of the AMF community, including spore morphological identification, DNA and RNA sequencing, examining the proteome, and mining genomes for the presence or absence of specific genes, such as effector proteins (Aparicio Chacón et al., 2023; Beaudet et al., 2018; Couto et al., 2013; Oehl et al., 2011; Venice et al., 2020; Victorino et al., 2020). For example, effector proteins which are important for establishing the initial symbiosis with the host plant may shed light on how AMF physiology alters plants, and it has been suggested that AMF may use effector proteins to their benefit (Aparicio Chacón et al., 2023). These experimental tools are useful to generate data on AMF community composition and diversity that allow researchers to observe patterns, yet challenges remain in the translation of these results to AMF function. To mitigate and work with these challenges, we think it is necessary to continue to intentionally disentangle the interplay between AMF and their hosts in natural systems to generate pertinent data for exploring the entirety of this symbiosis.

Taken together, our observations support the idea that increasing AMF diversity along a natural resource gradient coincides with increasing host–plant performance (AMF supports plant performance; Figure 1). The additive effect occurring within the AMF community that leads to an increase in diversity contributes to the understanding of this complex symbiosis by examining AMF community dynamics along this naturally occurring and highly stable resource gradient of soil N availability that also supports plant growth. These findings emphasize the need to further understand the bi‐directional interactions between AMF, the plant hosts, and their environments for a holistic understanding on their interplay in natural gradients of resource availability. Finally, these results naturally lead to questions that address the other side of the symbiosis by linking aboveground tree growth models to the belowground AMF community. Being able to connect the aboveground plant performance to the belowground mycorrhizal communities through this shared lens of soil mycorrhizal ecology and forest ecology, will shed light on how these communities impact each other throughout this complex symbiosis.

## FUNDING INFORMATION

This research was funded by the National Science Foundation (NSF grant DEB 2132002).

## CONFLICT OF INTEREST STATEMENT

None declared.

## Supporting information


Data S1:

## Data Availability

The data that support the findings of this study are openly available at NCBI and the code used for the microbial analyses is available on Zenodo (DOI: 10.5281/zenodo.13147393).

## References

[pei370002-bib-0001] Aparicio Chacón, M. V. , Van Dingenen, J. , & Goormachtig, S. (2023). Characterization of arbuscular mycorrhizal effector proteins. International Journal of Molecular Sciences, 24(11), 9125. 10.3390/ijms24119125 37298075 PMC10252856

[pei370002-bib-0002] Argiroff, W. A. , Zak, D. R. , Pellitier, P. T. , Upchurch, R. A. , & Belke, J. P. (2022). Decay by ectomycorrhizal fungi couples soil organic matter to nitrogen availability. Ecology Letters, 25(2), 391–404. 10.1111/ele.13923 34787356

[pei370002-bib-0003] Baker, M. E. , & King, R. S. (2010). A new method for detecting and interpreting biodiversity and ecological community thresholds: Threshold Indicator Taxa ANalysis (TITAN). Methods in Ecology and Evolution, 1(1), 25–37. 10.1111/j.2041-210X.2009.00007.x

[pei370002-bib-0004] Beaudet, D. , Chen, E. C. H. , Mathieu, S. , Yildirir, G. , Ndikumana, S. , Dalpé, Y. , Séguin, S. , Farinelli, L. , Stajich, J. E. , & Corradi, N. (2018). Ultra‐low input transcriptomics reveal the spore functional content and phylogenetic affiliations of poorly studied arbuscular mycorrhizal fungi. DNA Research, 25(2), 217–227. 10.1093/dnares/dsx051 29211832 PMC5909441

[pei370002-bib-0005] Bertolazi, A. A. , Da Silva Folli‐Pereira, M. , Caione, G. , Passamani, L. Z. , Colodete, C. M. , De Souza, S. B. , Ramos, A. C. , Rasool, N. , & Schoninger, E. L. (2018). Linking plant nutritional status to plant‐AMF interactions. In D. Egamberdieva & P. Ahmad (Eds.), Plant microbiome: Stress response (Vol. 5, pp. 351–384). Springer. 10.1007/978-981-10-5514-0_16

[pei370002-bib-0006] Bigelow, S. W. , Looney, C. E. , & Cannon, J. B. (2021). Hurricane effects on climate‐adaptive silviculture treatments to longleaf pine woodland in southwestern Georgia, USA. Forestry: An International Journal of Forest Research, 94(3), 395–406. 10.1093/forestry/cpaa042

[pei370002-bib-0007] Brundrett, M. C. , & Tedersoo, L. (2018). Evolutionary history of mycorrhizal symbioses and global host plant diversity. New Phytologist, 220(4), 1108–1115. 10.1111/nph.14976 29355963

[pei370002-bib-0008] Bücking, H. , & Kafle, A. (2015). Role of arbuscular mycorrhizal fungi in the nitrogen uptake of plants: Current knowledge and research gaps. Agronomy, 5(4), 587–612. 10.3390/agronomy5040587

[pei370002-bib-0009] Burns, R. , & Honkala, B. (1990). Silvis of North America, Hardwoods (Vol. 2). U.S. Department of Agruculture, Forest Service.

[pei370002-bib-0010] Callahan, B. J. , McMurdie, P. J. , & Holmes, S. P. (2017). Exact sequence variants should replace operational taxonomic units in marker‐gene data analysis. The ISME Journal, 11(12), 2639–2643. 10.1038/ismej.2017.119 28731476 PMC5702726

[pei370002-bib-0011] Callahan, B. J. , McMurdie, P. J. , Rosen, M. J. , Han, A. W. , Johnson, A. J. A. , & Holmes, S. P. (2016). DADA2: High‐resolution sample inference from Illumina amplicon data. Nature Methods, 13(7), 581–583. 10.1038/nmeth.3869 27214047 PMC4927377

[pei370002-bib-0012] Chao, A. (1984). Nonparametric estimation of the number of classes in a population. Scandinavian Journal of Statistics, 11, 265–270.

[pei370002-bib-0013] Chen, J. , Guo, J. , Li, Z. , Liang, X. , You, Y. , Li, M. , He, Y. , & Zhan, F. (2022). Effects of an arbuscular mycorrhizal fungus on the growth of and cadmium uptake in maize grown on polluted wasteland, farmland and slopeland soils in a lead‐zinc mining area. Toxics, 10(7), 359. 10.3390/toxics10070359 35878264 PMC9322003

[pei370002-bib-0014] Chitarra, W. , Pagliarani, C. , Maserti, B. , Lumini, E. , Siciliano, I. , Cascone, P. , Schubert, A. , Gambino, G. , Balestrini, R. , & Guerrieri, E. (2016). Insights on the impact of arbuscular mycorrhizal symbiosis on tomato tolerance to water stress. Plant Physiology, 171(2), 1009–1023. 10.1104/pp.16.00307 27208301 PMC4902612

[pei370002-bib-0015] Cook, E. R. (1990). Bootstrap confidence intervals for red spruce ring‐width chronologies and an assessment of age‐related bias in recent growth trends. Canadian Journal of Forest Research, 20(9), 1326–1331. 10.1139/x90-176

[pei370002-bib-0016] Couto, M. S. R. , Lovato, P. E. , Wipf, D. , & Dumas‐Gaudot, E. (2013). Proteomic studies of arbuscular mycorrhizal associations. Advances in Biological Chemistry, 3(1), 48–58. 10.4236/abc.2013.31007

[pei370002-bib-0017] Daniell, T. J. , Husband, R. , Fitter, A. H. , & Young, J. P. W. (2001). Molecular diversity of arbuscular mycorrhizal fungi colonising arable crops. FEMS Microbiology Ecology, 36(203), –209. 10.1111/j.1574-6941.2001.tb00841.x 11451525

[pei370002-bib-0018] Davison, J. , Öpik, M. , Daniell, T. J. , Moora, M. , & Zobel, M. (2011). Arbuscular mycorrhizal fungal communities in plant roots are not random assemblages: Selectivity in AMF‐plant associations. FEMS Microbiology Ecology, 78(1), 103–115. 10.1111/j.1574-6941.2011.01103.x 21457278

[pei370002-bib-0019] Davison, J. , Öpik, M. , Zobel, M. , Vasar, M. , Metsis, M. , & Moora, M. (2012). Communities of arbuscular mycorrhizal fungi detected in forest soil are spatially heterogeneous but do not vary throughout the growing season. PLoS One, 7(8), e41938. 10.1371/journal.pone.0041938 22879900 PMC3413688

[pei370002-bib-0020] Delavaux, C. S. , Smith‐Ramesh, L. M. , & Kuebbing, S. E. (2017). Beyond nutrients: A meta‐analysis of the diverse effects of arbuscular mycorrhizal fungi on plants and soils. Ecology, 98(8), 2111–2119. 10.1002/ecy.1892 28500779

[pei370002-bib-0021] Diagne, N. , Ngom, M. , Djighaly, P. I. , Fall, D. , Hocher, V. , & Svistoonoff, S. (2020). Roles of arbuscular mycorrhizal fungi on plant growth and performance: Importance in biotic and abiotic stressed regulation. Diversity, 12(10), 370. 10.3390/d12100370

[pei370002-bib-0022] Djotan, A. K. G. , Matsushita, N. , & Fukuda, K. (2023). Paired root‐soil samples and metabarcoding reveal taxon‐based colonization strategies in arbuscular mycorrhizal fungi communities in Japanese cedar and cypress stands. Microbial Ecology, 86(3), 2133–2146. 10.1007/s00248-023-02223-9 37115261 PMC10497666

[pei370002-bib-0023] Dumbrell, A. J. , Nelson, M. , Helgason, T. , Dytham, C. , & Fitter, A. H. (2010). Relative roles of niche and neutral processes in structuring a soil microbial community. The ISME Journal, 4(3), 337–345. 10.1038/ismej.2009.122 19924158

[pei370002-bib-0024] Duncan, R. P. (1989). An evaluation of errors in tree age estimates based on increment cores in kahikatea (*Dacrycarpus dacrydioides*). New Zealand Natural Sciences, 16, 31–37.

[pei370002-bib-0025] Egerton‐Warburton, L. M. , Johnson, N. C. , & Allen, E. B. (2007). Mycorrhizal community dynamics following nitrogen fertilization: A cross‐site test in five grasslands. Ecological Monographs, 77(4), 527–544. 10.1890/06-1772.1

[pei370002-bib-0026] Fellbaum, C. R. , Gachomo, E. W. , Beesetty, Y. , Choudhari, S. , Strahan, G. D. , Pfeffer, P. E. , Kiers, E. T. , & Bücking, H. (2012). Carbon availability triggers fungal nitrogen uptake and transport in arbuscular mycorrhizal symbiosis. Proceedings of the National Academy of Sciences, 109(7), 2666–2671. 10.1073/pnas.1118650109 PMC3289346 22308426

[pei370002-bib-0027] Fellbaum, C. R. , Mensah, J. A. , Cloos, A. J. , Strahan, G. E. , Pfeffer, P. E. , Kiers, E. T. , & Bücking, H. (2014). Fungal nutrient allocation in common mycorrhizal networks is regulated by the carbon source strength of individual host plants. New Phytologist, 203(2), 646–656. 10.1111/nph.12827 24787049

[pei370002-bib-0028] Hart, M. M. , & Reader, R. J. (2002). Taxonomic basis for variation in the colonization strategy of arbuscular mycorrhizal fungi. New Phytologist, 153(2), 335–344. 10.1046/j.0028-646X.2001.00312.x

[pei370002-bib-0029] Helgason, T. , Daniell, T. J. , Husband, R. , Fitter, A. H. , & Young, J. P. W. (1998). Ploughing up the wood‐wide web? Nature, 394(6692), 431. 10.1038/28764 9697763

[pei370002-bib-0030] Hiiesalu, I. , Pärtel, M. , Davison, J. , Gerhold, P. , Metsis, M. , Moora, M. , Öpik, M. , Vasar, M. , Zobel, M. , & Wilson, S. D. (2014). Species richness of arbuscular mycorrhizal fungi: Associations with grassland plant richness and biomass. New Phytologist, 203(1), 233–244. 10.1111/nph.12765 24641509

[pei370002-bib-0031] Hodge, A. , & Fitter, A. H. (2010). Substantial nitrogen acquisition by arbuscular mycorrhizal fungi from organic material has implications for N cycling. Proceedings of the National Academy of Sciences, 107(31), 13754–13759. 10.1073/pnas.1005874107 PMC2922220 20631302

[pei370002-bib-0032] Hughes, J. B. , Hellmann, J. J. , Ricketts, T. H. , & Bohannan, B. J. M. (2001). Counting the uncountable: Statistical approaches to estimating microbial diversity. Applied and Environmental Microbiology, 67(10), 4399–4406. 10.1128/AEM.67.10.4399-4406.2001 11571135 PMC93182

[pei370002-bib-0033] Ibáñez, I. , & McCarthy‐Neumann, S. (2016). Effects of mycorrhizal fungi on tree seedling growth: Quantifying the parasitism–mutualism transition along a light gradient. Canadian Journal of Forest Research, 46(1), 48–57. 10.1139/cjfr-2015-0327

[pei370002-bib-0034] Ibáñez, I. , Zak, D. R. , Burton, A. J. , & Pregitzer, K. S. (2018). Anthropogenic nitrogen deposition ameliorates the decline in tree growth caused by a drier climate. Ecology, 99(2), 411–420. 10.1002/ecy.2095 29341107

[pei370002-bib-0035] Johnson, N. C. , Rowland, D. L. , Corkidi, L. , Egerton‐Warburton, L. M. , & Allen, E. B. (2003). Nitrogen enrichment alters mycorrhizal allocation at five mesic to semiarid grasslands. Ecology, 84(7), 1895–1908. 10.1890/0012-9658(2003)084[1895:NEAMAA]2.0.CO;2

[pei370002-bib-0036] Katoh, K. (2002). MAFFT: A novel method for rapid multiple sequence alignment based on fast Fourier transform. Nucleic Acids Research, 30(14), 3059–3066. 10.1093/nar/gkf436 12136088 PMC135756

[pei370002-bib-0037] Kim, B.‐R. , Shin, J. , Guevarra, R. B. , Lee, J. H. , Kim, D. W. , Seol, K.‐H. , Lee, J.‐H. , Kim, H. B. , & Isaacson, R. E. (2017). Deciphering diversity indices for a better understanding of microbial communities. Journal of Microbiology and Biotechnology, 27(12), 2089–2093. 10.4014/jmb.1709.09027 29032640

[pei370002-bib-0501] Kokkoris, V. , Banchini, C. , Paré, L. , Abdellatif, L. , Séguin, S. , Hubbard, K. , Findlay, W. , Dalpé, Y. , Dettman, J. , Corradi, N. , & Stefani, F. (2024). *Rhizophagus irregularis*, the model fungus in arbuscular mycorrhiza research, forms dimorphic spores. New Phytologist, 242(4), 1771–1784. 10.1111/nph.19121 37434339

[pei370002-bib-0038] Lanfranco, L. , Bonfante, P. , & Genre, A. (2016). The mutualistic interaction between plants and arbuscular mycorrhizal fungi. Microbiology Spectrum, 4(6), 10. 10.1128/microbiolspec.FUNK-0012-2016 28087942

[pei370002-bib-0039] Lee, J. , Lee, S. , & Young, J. P. W. (2008). Improved PCR primers for the detection and identification of arbuscular mycorrhizal fungi: PCR primers for arbuscular mycorrhizal fungi. FEMS Microbiology Ecology, 65(2), 339–349. 10.1111/j.1574-6941.2008.00531.x 18631176

[pei370002-bib-0504] Legendre, P. , & Legendre, L. (2012). Numerical ecology. Elsevier.

[pei370002-bib-0040] Lekberg, Y. , Koide, R. T. , Rohr, J. R. , Aldrich‐Wolfe, L. , & Morton, J. B. (2007). Role of niche restrictions and dispersal in the composition of arbuscular mycorrhizal fungal communities. Journal of Ecology, 95(1), 95–105. 10.1111/j.1365-2745.2006.01193.x

[pei370002-bib-0041] Letunic, I. , & Bork, P. (2021). Interactive Tree Of Life (iTOL) v5: An online tool for phylogenetic tree display and annotation. Nucleic Acids Research, 49(W1), W293–W296. 10.1093/nar/gkab301 33885785 PMC8265157

[pei370002-bib-0505] Lines, E. R. , Zavala, M. A. , Purves, D. W. , & Coomes, D. A. (2012). Predictable changes in aboveground allometry of trees along gradients of temperature, aridity and competition. Global Ecology and Biogeography, 21(10), 1017–1028. 10.1111/j.1466-8238.2011.00746.x

[pei370002-bib-0042] Liu, M. , Shen, Y. , Li, Q. , Xiao, W. , & Song, X. (2021). Arbuscular mycorrhizal fungal colonization and soil pH induced by nitrogen and phosphorus additions affects leaf C:N:P stoichiometry in Chinese fir (*Cunninghamia lanceolata*) forests. Plant and Soil, 461(1–2), 421–440. 10.1007/s11104-021-04831-1

[pei370002-bib-0043] Ma, X. , Geng, Q. , Zhang, H. , Bian, C. , Chen, H. Y. H. , Jiang, D. , & Xu, X. (2021). Global negative effects of nutrient enrichment on arbuscular mycorrhizal fungi, plant diversity and ecosystem multifunctionality. New Phytologist, 229(5), 2957–2969. 10.1111/nph.17077 33188641

[pei370002-bib-0044] Martin, M. (2011). Cutadapt removes adapter sequences from high‐throughput sequencing reads. EMBnet.Journal, 17(1), 10. 10.14806/ej.17.1.200

[pei370002-bib-0045] McMurdie, P. J. , & Holmes, S. (2013). phyloseq: An R package for reproducible interactive analysis and graphics of microbiome census data. PLoS One, 8(4), e61217. 10.1371/journal.pone.0061217 23630581 PMC3632530

[pei370002-bib-0046] Morgan, B. S. T. , & Egerton‐Warburton, L. M. (2017). Barcoded NS31/AML2 primers for sequencing of arbuscular mycorrhizal communities in environmental samples. Applications in Plant Sciences, 5(8), 1700017. 10.3732/apps.1700017 PMC5584815 28924511

[pei370002-bib-0047] Nakmee, P. S. , Techapinyawat, S. , & Ngamprasit, S. (2016). Comparative potentials of native arbuscular mycorrhizal fungi to improve nutrient uptake and biomass of *Sorghum bicolor* Linn. Agriculture and Natural Resources, 50(3), 173–178. 10.1016/j.anres.2016.06.004

[pei370002-bib-0048] Oehl, F. , Sieverding, E. , Palenzuela, J. , Ineichen, K. , & Da Silva, G. A. (2011). Advances in Glomeromycota taxonomy and classification. IMA Fungus, 2(2), 191–199. 10.5598/imafungus.2011.02.02.10 22679604 PMC3359817

[pei370002-bib-0049] Oksanen, J. , Simpson, G. L. , Blanchet, F. G. , Kindt, R. , Legendre, P. , Minchin, P. R. , O'Hara, R. B. , Solymos, P. , Stevens, M. H. H. , Szoecs, E. , Wagner, H. , Barbour, M. , Bedward, M. , Bolker, B. , Borcard, D. , Carvalho, G. , Chirico, M. , Caceres, M. D. , Durand, S. , & Weedon, J. (2022). vegan: Community Ecology Package [Computer software]. https://CRAN.R‐project.org/package=vegan

[pei370002-bib-0050] Öpik, M. , Vanatoa, A. , Vanatoa, E. , Moora, M. , Davison, J. , Kalwij, J. M. , Reier, Ü. , & Zobel, M. (2010). The online database Maarj AM reveals global and ecosystemic distribution patterns in arbuscular mycorrhizal fungi (Glomeromycota). New Phytologist, 188(1), 223–241. 10.1111/j.1469-8137.2010.03334.x 20561207

[pei370002-bib-0051] Ordoñez, J. C. , Van Bodegom, P. M. , Witte, J. M. , Wright, I. J. , Reich, P. B. , & Aerts, R. (2009). A global study of relationships between leaf traits, climate and soil measures of nutrient fertility. Global Ecology and Biogeography, 18(2), 137–149. 10.1111/j.1466-8238.2008.00441.x

[pei370002-bib-0052] Pagès, H. , Aboyoun, P. , Gentleman, R. , & DebRoy, S. (2020). Biostrings: Efficient manipulation of biological strings [Computer software]. https://bioconductor.org/packages/Biostrings

[pei370002-bib-0053] Pauvert, C. , Buée, M. , Laval, V. , Edel‐Hermann, V. , Fauchery, L. , Gautier, A. , Lesur, I. , Vallance, J. , & Vacher, C. (2019). Bioinformatics matters: The accuracy of plant and soil fungal community data is highly dependent on the metabarcoding pipeline. Fungal Ecology, 41, 23–33. 10.1016/j.funeco.2019.03.005

[pei370002-bib-0054] Pellitier, P. T. , Ibáñez, I. , Zak, D. R. , Argiroff, W. A. , & Acharya, K. (2021). Ectomycorrhizal access to organic nitrogen mediates CO_2_ fertilization response in a dominant temperate tree. Nature Communications, 12(1), 5403. 10.1038/s41467-021-25652-x PMC8438073 34518539

[pei370002-bib-0055] Pellitier, P. T. , Zak, D. R. , Argiroff, W. A. , & Upchurch, R. A. (2021). Coupled shifts in ectomycorrhizal communities and plant uptake of organic nitrogen along a soil gradient: An isotopic perspective. Ecosystems, 24(8), 1976–1990. 10.1007/s10021-021-00628-6

[pei370002-bib-0056] Plummer, M. (2021). JAGS: A Program for Analysis of Bayesian Graphical Models using Gibbs Sampling [Computer software]. https://sourceforge.net/projects/mcmc‐jags/

[pei370002-bib-0057] Posit Team . (2023). RStudio: Integrated development environment for R [Computer software]. Posit Software, PBC.

[pei370002-bib-0058] R Core Team . (2023). R: A language and environment for statistical computing [Computer software]. R Foundation for Statistical Computing.

[pei370002-bib-0059] Reich, P. B. , Hobbie, S. E. , Lee, T. , Ellsworth, D. S. , West, J. B. , Tilman, D. , Knops, J. M. H. , Naeem, S. , & Trost, J. (2006). Nitrogen limitation constrains sustainability of ecosystem response to CO_2_ . Nature, 440(7086), 922–925. 10.1038/nature04486 16612381

[pei370002-bib-0060] Rennenberg, H. , Dannenmann, M. , Gessler, A. , Kreuzwieser, J. , Simon, J. , & Papen, H. (2009). Nitrogen balance in forest soils: Nutritional limitation of plants under climate change stresses. Plant Biology, 11(s1), 4–23. 10.1111/j.1438-8677.2009.00241.x 19778364

[pei370002-bib-0061] Rosen, M. J. , Callahan, B. J. , Fisher, D. S. , & Holmes, S. P. (2012). Denoising PCR‐amplified metagenome data. BMC Bioinformatics, 13(1), 283. 10.1186/1471-2105-13-283 23113967 PMC3563472

[pei370002-bib-0062] Roth, R. , & Paszkowski, U. (2017). Plant carbon nourishment of arbuscular mycorrhizal fungi. Current Opinion in Plant Biology, 39, 50–56. 10.1016/j.pbi.2017.05.008 28601651

[pei370002-bib-0063] Simon, L. , Lalonde, M. , & Bruns, T. D. (1992). Specific amplification of 18S fungal ribosomal genes from vesicular‐arbuscular endomycorrhizal fungi colonizing roots. Applied and Environmental Microbiology, 58(1), 291–295. 10.1128/aem.58.1.291-295.1992 1339260 PMC195206

[pei370002-bib-0064] Smith, S. E. , Jakobsen, I. , Grønlund, M. , & Smith, F. A. (2011). Roles of arbuscular mycorrhizas in plant phosphorus nutrition: Interactions between pathways of phosphorus uptake in arbuscular mycorrhizal roots have important implications for understanding and manipulating plant phosphorus acquisition. Plant Physiology, 156(3), 1050–1057. 10.1104/pp.111.174581 21467213 PMC3135927

[pei370002-bib-0065] Smith, S. E. , & Read, D. J. (2008). Mycorrhizal symbiosis (3rd ed.). Academic Press.

[pei370002-bib-0066] Spiegelhalter, D. J. , Best, N. G. , Carlin, B. P. , & Van Der Linde, A. (2002). Bayesian measures of model complexity and fit. Journal of the Royal Statistical Society, Series B: Statistical Methodology, 64(4), 583–639. 10.1111/1467-9868.00353

[pei370002-bib-0502] Sugiura, Y. , Akiyama, R. , Tanaka, S. , Yano, K. , Kameoka, H. , Marui, S. , Saito, M. , Kawaguchi, M. , Akiyama, K. , & Saito, K. (2020). Myristate can be used as a carbon and energy source for the asymbiotic growth of arbuscular mycorrhizal fungi. Proceedings of the National Academy of Sciences of the United States of America, 117(41), 25779–25788. 10.1073/pnas.2006948117 32999061 PMC7568319

[pei370002-bib-0067] Suzuki, K. , Takahashi, K. , & Harada, N. (2020). Evaluation of primer pairs for studying arbuscular mycorrhizal fungal community compositions using a MiSeq platform. Biology and Fertility of Soils, 56(6), 853–858. 10.1007/s00374-020-01431-6

[pei370002-bib-0068] Tamura, K. , Stecher, G. , & Kumar, S. (2021). MEGA11: Molecular evolutionary genetics analysis version 11. Molecular Biology and Evolution, 38(7), 3022–3027. 10.1093/molbev/msab120 33892491 PMC8233496

[pei370002-bib-0069] Taylor, D. L. , Walters, W. A. , Lennon, N. J. , Bochicchio, J. , Krohn, A. , Caporaso, J. G. , & Pennanen, T. (2016). Accurate estimation of fungal diversity and abundance through improved lineage‐specific primers optimized for illumina amplicon sequencing. Applied and Environmental Microbiology, 82(24), 7217–7226. 10.1128/AEM.02576-16 27736792 PMC5118932

[pei370002-bib-0503] Terrer, C. , Vicca, S. , Stocker, B. D. , Hungate, B. A. , Phillips, R. P. , Reich, P. B. , Finzi, A. C. , & Prentice, I. C. (2018). Ecosystem responses to elevated CO_2_ governed by plant–soil interactions and the cost of nitrogen acquisition. New Phytologist, 217(2), 507–522. 10.1111/nph.14872 29105765

[pei370002-bib-0070] Terrer, C. , Jackson, R. B. , Prentice, I. C. , Keenan, T. F. , Kaiser, C. , Vicca, S. , Fisher, J. B. , Reich, P. B. , Stocker, B. D. , Hungate, B. A. , Peñuelas, J. , McCallum, I. , Soudzilovskaia, N. A. , Cernusak, L. A. , Talhelm, A. F. , Van Sundert, K. , Piao, S. , Newton, P. C. D. , Hovenden, M. J. , & Franklin, O. (2019). Nitrogen and phosphorus constrain the CO_2_ fertilization of global plant biomass. Nature Climate Change, 9(9), 684–689. 10.1038/s41558-019-0545-2

[pei370002-bib-0071] Treseder, K. K. , & Allen, M. F. (2002). Direct nitrogen and phosphorus limitation of arbuscular mycorrhizal fungi: A model and field test. New Phytologist, 155(3), 507–515. 10.1046/j.1469-8137.2002.00470.x 33873310

[pei370002-bib-0072] Van Der Heijden, M. G. A. , Klironomos, J. N. , Ursic, M. , Moutoglis, P. , Streitwolf‐Engel, R. , Boller, T. , Wiemken, A. , & Sanders, I. R. (1998). Mycorrhizal fungal diversity determines plant biodiversity, ecosystem variability and productivity. Nature, 396(6706), 69–72. 10.1038/23932

[pei370002-bib-0073] Van Der Heijden, M. G. A. , Martin, F. M. , Selosse, M. , & Sanders, I. R. (2015). Mycorrhizal ecology and evolution: The past, the present, and the future. New Phytologist, 205(4), 1406–1423. 10.1111/nph.13288 25639293

[pei370002-bib-0074] Van Diepen, L. T. A. , Lilleskov, E. A. , & Pregitzer, K. S. (2011). Simulated nitrogen deposition affects community structure of arbuscular mycorrhizal fungi in northern hardwood forests: Nitrogen effects on AMF community structure. Molecular Ecology, 20(4), 799–811. 10.1111/j.1365-294X.2010.04969.x 21210962

[pei370002-bib-0075] Venice, F. , Ghignone, S. , Salvioli Di Fossalunga, A. , Amselem, J. , Novero, M. , Xianan, X. , Sędzielewska Toro, K. , Morin, E. , Lipzen, A. , Grigoriev, I. V. , Henrissat, B. , Martin, F. M. , & Bonfante, P. (2020). At the nexus of three kingdoms: The genome of the mycorrhizal fungus *Gigaspora margarita* provides insights into plant, endobacterial and fungal interactions. Environmental Microbiology, 22(1), 122–141. 10.1111/1462-2920.14827 31621176

[pei370002-bib-0076] Větrovský, T. , Kolaříková, Z. , Lepinay, C. , Awokunle Hollá, S. , Davison, J. , Fleyberková, A. , Gromyko, A. , Jelínková, B. , Kolařík, M. , Krüger, M. , Lejsková, R. , Michalčíková, L. , Michalová, T. , Moora, M. , Moravcová, A. , Moulíková, Š. , Odriozola, I. , Öpik, M. , Pappová, M. , & Kohout, P. (2023). globalamfungi: A global database of arbuscular mycorrhizal fungal occurrences from high‐throughput sequencing metabarcoding studies. New Phytologist, 240(5), 2151–2163. 10.1111/nph.19283 37781910

[pei370002-bib-0077] Victorino, Í. M. M. , Berruti, A. , Orgiazzi, A. , Voyron, S. , Bianciotto, V. , & Lumini, E. (2020). High‐throughput DNA sequence‐based analysis of AMF communities. In N. Ferrol & L. Lanfranco (Eds.), Arbuscular mycorrhizal fungi (Vol. 2146, pp. 99–116). Springer. 10.1007/978-1-0716-0603-2_9 32415599

[pei370002-bib-0078] Vitousek, P. M. , Gosz, J. R. , Grier, C. C. , Melillo, J. M. , & Reiners, W. A. (1982). A comparative analysis of potential nitrification and nitrate mobility in forest ecosystems. Ecological Monographs, 52(2), 155–177. 10.2307/1942609

[pei370002-bib-0079] Wang, J. , Zhang, J. , Wang, C. , Ren, G. , Yang, Y. , & Wang, D. (2021). Precipitation exerts a strong influence on arbuscular mycorrhizal fungi community and network complexity in a semiarid steppe ecosystem. European Journal of Soil Biology, 102, 103268. 10.1016/j.ejsobi.2020.103268

[pei370002-bib-0080] Wang, M. , Wang, Z. , Guo, M. , Qu, L. , & Biere, A. (2023). Effects of arbuscular mycorrhizal fungi on plant growth and herbivore infestation depend on availability of soil water and nutrients. Frontiers in Plant Science, 14, 1101932. 10.3389/fpls.2023.1101932 36778709 PMC9909235

[pei370002-bib-0081] Wang, X. , & Ibáñez, I. (2022). The contrasting effects of local environmental conditions on tree growth between populations at different latitudes. Forests, 13(3), 429. 10.3390/f13030429

[pei370002-bib-0082] Wickham, H. , Averick, M. , Bryan, J. , Chang, W. , McGowan, L. , François, R. , Grolemund, G. , Hayes, A. , Henry, L. , Hester, J. , Kuhn, M. , Pedersen, T. , Miller, E. , Bache, S. , Müller, K. , Ooms, J. , Robinson, D. , Seidel, D. , Spinu, V. , & Yutani, H. (2019). Welcome to the Tidyverse. Journal of Open Source Software, 4(43), 1686. 10.21105/joss.01686

[pei370002-bib-0083] Wieder, W. R. , Allison, S. D. , Davidson, E. A. , Georgiou, K. , Hararuk, O. , He, Y. , Hopkins, F. , Luo, Y. , Smith, M. J. , Sulman, B. , Todd‐Brown, K. , Wang, Y. , Xia, J. , & Xu, X. (2015). Explicitly representing soil microbial processes in Earth system models. Global Biogeochemical Cycles, 29(10), 1782–1800. 10.1002/2015GB005188

[pei370002-bib-0084] Xie, K. , Ren, Y. , Chen, A. , Yang, C. , Zheng, Q. , Chen, J. , Wang, D. , Li, Y. , Hu, S. , & Xu, G. (2022). Plant nitrogen nutrition: The roles of arbuscular mycorrhizal fungi. Journal of Plant Physiology, 269, 153591. 10.1016/j.jplph.2021.153591 34936969

[pei370002-bib-0085] Zak, D. R. , Host, G. E. , & Pregitzer, K. S. (1989). Regional variability in nitrogen mineralization, nitrification, and overstory biomass in northern Lower Michigan. Canadian Journal of Forest Research, 19(12), 1521–1526. 10.1139/x89-231

[pei370002-bib-0086] Zak, D. R. , & Pregitzer, K. S. (1990). Spatial and temporal variability of nitrogen cycling in northern lower Michigan. Forest Science, 36, 367–380. 10.1093/forestscience/36.2.367

[pei370002-bib-0087] Zak, D. R. , Pregitzer, K. S. , & Host, G. E. (1986). Landscape variation in nitrogen mineralization and nitrification. Canadian Journal of Forest Research, 16(6), 1258–1263. 10.1139/x86-223

