# Supplementary material for: Arbuscular mycorrhizal diversity increases across a plant productivity gradient driven by soil nitrogen availability
Source: Plant Environ Interact. 2024 Aug 10;5(4):e70002. doi: 10.1002/pei3.70002 (PMC11316137; doi:10.1002/pei3.70002)
Supplement: Supplementary file 1 — Data S1: [file PEI3-5-e70002-s001.docx]

## Plant-Environment Interactions Supporting Information

**Article title:** Arbuscular mycorrhizal diversity increases across a plant productivity gradient driven by soil nitrogen availability

**Authors:** Morgan R. McPherson, Donald R. Zak, Inés Ibáñez, Rima A. Upchurch, William A. Argiroff

**The following Supporting Information is available for this article:**

Fig. S1. Location of the 12 study sites that span the N mineralization gradient in northern Lower Michigan, USA.

Fig. S2. Phylogenetic tree of representative Glomeromycota inclusive of ASV reference sequences from our dataset, VTs assigned to the ASVs, and at least one example from other genera represented in the MaarjAM database.

Table S1. Primers and sequencing information

Table S2. Soil environmental data

Table S3. Parameter values, means, SDs, and 95% credible intervals, from the tree growth analysis.

Fig. S1. Location of the 12 study sites that span the N mineralization gradient in northern Lower Michigan, USA (Argiroff et al., 2022). Note that ‘stand’ and ‘site’ are interchangeable. More site details in Table S2 and in the metadata on Zenodo.


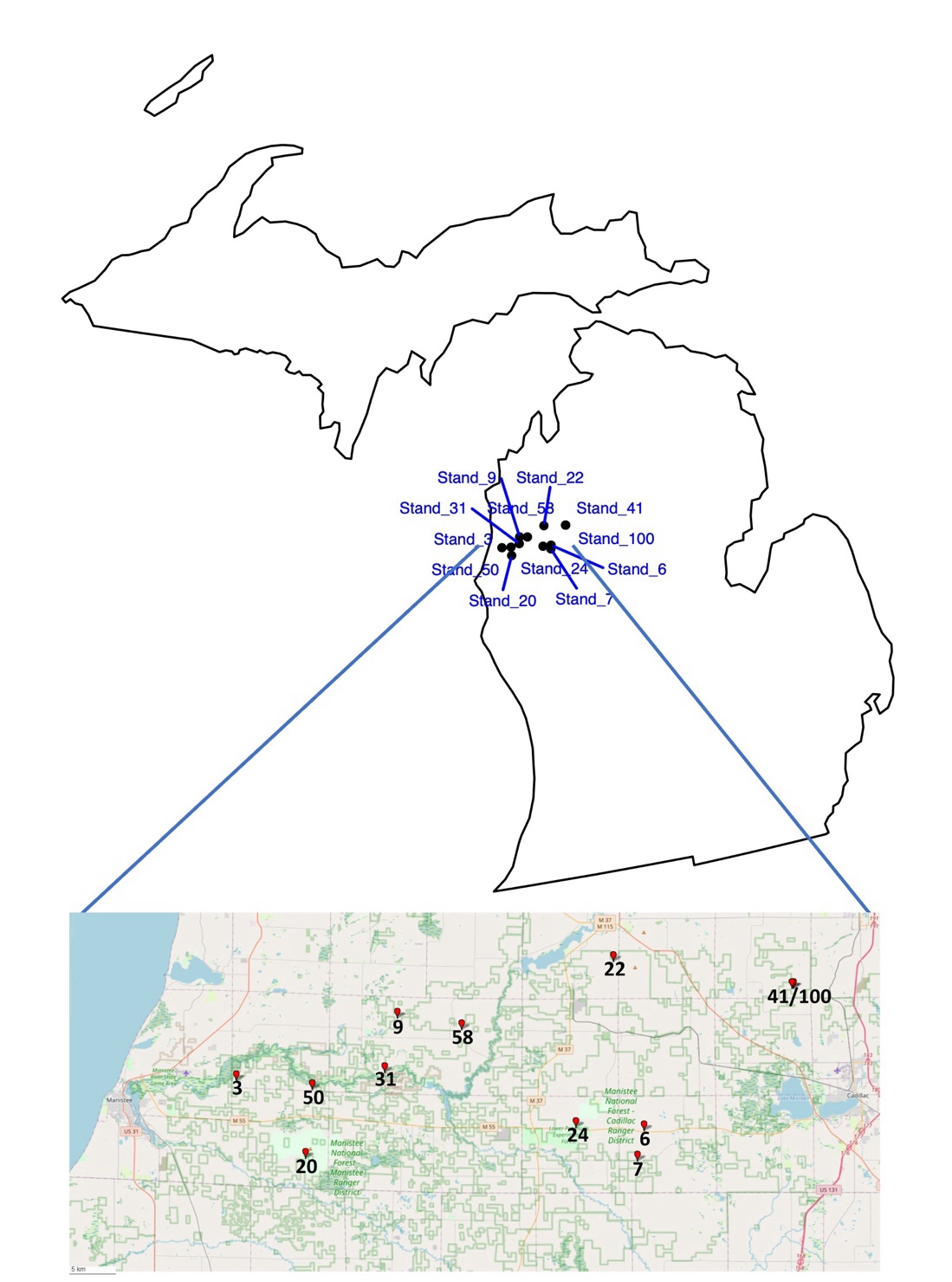


Fig. S2. Phylogenetic tree of representative Glomeromycota inclusive of ASV reference sequences from our dataset, VTs assigned to the ASVs, and at least one example from other genera represented in the MaarjAM database. Sequences were aligned and trimmed for quality, and the tree was created using MEGA software with 1000 bootstrap replicates. The AMF families are colored coded in the tree, with each color representing a family, and color ranges representing an order (see key). Shades of green are inclusive of the Archaeosporales order, yellow represents Paragrlomerales, shades of blue are Diversisporales, and shades of red encompass the Glomerales. All ASV reference sequences from our dataset are white.

Table S1. Primers and sequencing information

| Illumina sequencing adapter (i5 or i7), 8-bp Illumina index, 10-bp pad sequence, 2-bp linker sequence, and the NS31 (F) and AML2 (R) primers. | | |
| --- | --- | --- |
| *NS31* | TTGGAGGGCAAGTCTGGTGCC |  |
| 5' Illumina adapter i5 | AATGATACGGCGACCACCGAGATCTACAC |  |
| 8-bp Illumia index | see below |  |
| 10-bp pad | TATGGTAATT |  |
| 2-bp linker | AA |  |
| *Forward primers (5'-3')* | *Barcode* | *Full sequence* |
| SA502 | ACTATCTG | AATGATACGGCGACCACCGAGATCTACACACTATCTGTATGGTAATTAATTGGAGGGCAAGTCTGGTGCC |
| SA503 | TAGCGAGT | AATGATACGGCGACCACCGAGATCTACACTAGCGAGTTATGGTAATTAATTGGAGGGCAAGTCTGGTGCC |
| SA505 | TCATCGAG | AATGATACGGCGACCACCGAGATCTACACTCATCGAGTATGGTAATTAATTGGAGGGCAAGTCTGGTGCC |
| SA507 | GGATATCT | AATGATACGGCGACCACCGAGATCTACACGGATATCTTATGGTAATTAATTGGAGGGCAAGTCTGGTGCC |
| SA508 | GACACCGT | AATGATACGGCGACCACCGAGATCTACACGACACCGTTATGGTAATTAATTGGAGGGCAAGTCTGGTGCC |
| SB501 | CTACTATA | AATGATACGGCGACCACCGAGATCTACACCTACTATATATGGTAATTAATTGGAGGGCAAGTCTGGTGCC |
| SB503 | AGAGTCAC | AATGATACGGCGACCACCGAGATCTACACAGAGTCACTATGGTAATTAATTGGAGGGCAAGTCTGGTGCC |
| SB504 | TACGAGAC | AATGATACGGCGACCACCGAGATCTACACTACGAGACTATGGTAATTAATTGGAGGGCAAGTCTGGTGCC |
| SB506 | TCGACGAG | AATGATACGGCGACCACCGAGATCTACACTCGACGAGTATGGTAATTAATTGGAGGGCAAGTCTGGTGCC |
| SB508 | GTCAGATA | AATGATACGGCGACCACCGAGATCTACACGTCAGATATATGGTAATTAATTGGAGGGCAAGTCTGGTGCC |
|  |  |  |
| *AML2* | GAACCCAAACACTTTGGTTTCC |  |
| 5' Illumina adapter i7 | CAAGCAGAAGACGGCATACGAGAT |  |
| 8-bp Illumia index | see below |  |
| 10-bp pad | AGTCAGTCAG |  |
| 2-bp linker | GG |  |
| *Reverse primers (5'-3')* | *Barcode* | *Full sequence* |
| SA702 | ACTATGTC | CAAGCAGAAGACGGCATACGAGATACTATGTCAGTCAGTCAGGGGAACCCAAACACTTTGGTTTCC |
| SA704 | CAGTGAGT | CAAGCAGAAGACGGCATACGAGATCAGTGAGTAGTCAGTCAGGGGAACCCAAACACTTTGGTTTCC |
| SA707 | GGAGACTA | CAAGCAGAAGACGGCATACGAGATGGAGACTAAGTCAGTCAGGGGAACCCAAACACTTTGGTTTCC |
| SA710 | TAGCAGAC | CAAGCAGAAGACGGCATACGAGATTAGCAGACAGTCAGTCAGGGGAACCCAAACACTTTGGTTTCC |
| SB701 | AAGTCGAG | CAAGCAGAAGACGGCATACGAGATAAGTCGAGAGTCAGTCAGGGGAACCCAAACACTTTGGTTTCC |
| SB702 | ATACTTCG | CAAGCAGAAGACGGCATACGAGATATACTTCGAGTCAGTCAGGGGAACCCAAACACTTTGGTTTCC |
| SB703 | AGCTGCTA | CAAGCAGAAGACGGCATACGAGATAGCTGCTAAGTCAGTCAGGGGAACCCAAACACTTTGGTTTCC |
| SB704 | CATAGAGA | CAAGCAGAAGACGGCATACGAGATCATAGAGAAGTCAGTCAGGGGAACCCAAACACTTTGGTTTCC |
| SB708 | GGTACTAT | CAAGCAGAAGACGGCATACGAGATGGTACTATAGTCAGTCAGGGGAACCCAAACACTTTGGTTTCC |
| SB710 | TACGAGCA | CAAGCAGAAGACGGCATACGAGATTACGAGCAAGTCAGTCAGGGGAACCCAAACACTTTGGTTTCC |
| SB711 | TCAGCGTT | CAAGCAGAAGACGGCATACGAGATTCAGCGTTAGTCAGTCAGGGGAACCCAAACACTTTGGTTTCC |
|  |  |  |
| Sequencing primes |  |  |
| AMF_Read1_LNA | TAT+GG+TAATTAATTGGAGGGCAAGTCTGGTGCC | |
| AMF_Read2 | AGTCAGTCAGGGGAACCCAAACACTTTGGTTTCC | |
| AMF_Index | GGAAACCAAAGTGTTTGGGTTCCCCTGACTGACT | |
| *the position of the LNA is denoted by placing a "+" in front of the modified base | | |

Table S2. Soil environmental data: Site averages of latitude and longitude of all trees in the site, BAI of all the trees in a site across all 41 years, soil pH, net N mineralization rate, percentage of N, percentage of C, and percentage of soil moisture. Individual data for each sample can be found in the metadata on Zenodo.

| Site | Avg. Latitude | Avg. Longitude | Avg. BAI | Avg. N | Avg. soil pH | Avg. % N | Avg. % C | % soil moisture |
| --- | --- | --- | --- | --- | --- | --- | --- | --- |
|  |  |  |  | mineralization |  |  |  |  |
| 3 | 44.26415582 | -86.17795378 | 2.01 | 0.7 | 3.69 | 0.16 | 4 | 9.64 |
| 6 | 44.2198641 | -85.66850176 | 15.45 | 0.47 | 5.46 | 0.15 | 2.07 | 18.61 |
| 7 | 44.19263836 | -85.67795255 | 5.83 | 0.86 | 4.25 | 0.15 | 2.7 | 9.34 |
| 9 | 44.32029192 | -85.97698 | 6.11 | 0.72 | 4.03 | 0.12 | 2.42 | 13.07 |
| 20 | 44.19483112 | -86.0913715 | 4.48 | 0.66 | 3.62 | 0.15 | 3.52 | 8.45 |
| 22 | 44.37322684 | -85.70831864 | 14.19 | 1.56 | 4.35 | 0.17 | 2.47 | 12.32 |
| 24 | 44.22227095 | -85.75187814 | 12.71 | 1.25 | 4.51 | 0.17 | 2.52 | 16.08 |
| 31 | 44.27203236 | -85.99245216 | 6.11 | 1.21 | 3.8 | 0.15 | 3.5 | 17.25 |
| 41 | 44.34688783 | -85.48180105 | 9.76 | 1.36 | 3.75 | 0.13 | 1.88 | 11.65 |
| 50 | 44.25659468 | -86.08361476 | 2.19 | 0.36 | 3.6 | 0.16 | 3.83 | 6.75 |
| 58 | 44.310452 | -85.8967945 | 2.05 | 0.94 | 3.67 | 0.12 | 2.33 | 15.45 |
| 100 | 44.34707284 | -85.4833218 | 6.86 | 1.47 | 4.39 | 0.16 | 2.14 | 14.72 |

Table S3. Parameter values, means, SDs, and 95% credible intervals, from the tree growth analysis.

| ***Acer rubrum*** |  |  |  |  |  |
| --- | --- | --- | --- | --- | --- |
|  |  | mean | SD | 2.50% | 97.50% |
| Intercept | α | 0.580 | 0.278 | 0.044 | 0.979 |
| N mineralization | β_1_ | 1.061 | 0.261 | 0.529 | 1.536 |
| BAI2t-1 | β_2_ | 0.225 | 0.010 | 0.205 | 0.244 |
| MinMayTemp | β_4_ | 0.019 | 0.005 | 0.008 | 0.029 |
| ln(DBH) | β_3_ | -0.145 | 0.112 | -0.353 | 0.086 |
| Variance intercept | a | 0.290 | 0.041 | 0.209 | 0.369 |
| Variance slope ln(DBH) | b | -0.043 | 0.013 | -0.068 | -0.018 |
| Decay exponent spatial effect | ϕ | 1.659 | 2.165 | 0.139 | 7.141 |
| Variance spatial effects | σ^2^_SERE_ | 0.141 | 0.039 | 0.083 | 0.234 |
|  |  |  |  |  |  |
| ***Acer saccharum*** |  |  |  |  |  |
|  |  | mean | SD | 2.50% | 97.50% |
| Intercept | α | 0.860 | 0.136 | 0.541 | 0.996 |
| N mineralization | β_1_ | 1.829 | 0.909 | 0.106 | 3.138 |
| BAI2t-1 | β_2_ | 0.172 | 0.009 | 0.153 | 0.191 |
| MinMayTemp | β_4_ | -0.005 | 0.006 | -0.016 | 0.006 |
| ln(DBH) | β_3_ | -0.238 | 0.272 | -0.594 | 0.350 |
| Variance intercept | a | 0.000 | 0.000 | 0.000 | 0.000 |
| Variance slope ln(DBH) | b | 0.024 | 0.001 | 0.022 | 0.026 |
| Decay exponent spatial effect | ϕ | 1.597 | 1.969 | 0.136 | 6.504 |
| Variance spatial effects | σ^2^_SERE_ | 0.377 | 0.246 | 0.045 | 0.943 |

**References**

Argiroff, W. A., Zak, D. R., Pellitier, P. T., Upchurch, R. A., & Belke, J. P. (2022). Decay by ectomycorrhizal fungi couples soil organic matter to nitrogen availability. *Ecology Letters*, *25*(2), 391–404. https://doi.org/10.1111/ele.13923
